# Mouse tumor susceptibility genes identify drug combinations for multiple myeloma

**DOI:** 10.20517/2394-4722.2020.40

**Published:** 2020-07-26

**Authors:** Shuling Zhang, Wendy DuBois, Ke Zhang, John K. Simmons, V. Keith Hughitt, Sayeh Gorjifard, Snehal Gaikwad, Tyler J. Peat, Beverly A. Mock

**Affiliations:** 1 Laboratory of Cancer Biology and Genetics, CCR, NCI, NIH, Bethesda, MD 20892, USA; 2 Personal Genome Diagnostics, Baltimore, MD 21224, USA; 3 University of Washington School of Medicine, Department of Genome Sciences, Seattle, WA 98195, USA

**Keywords:** Complex genetic trait, plasma cell tumor, multiple myeloma, entinostat, rapamycin, drug combinations, *Cdkn2a*, *Mtor*, *Mndal*, *MYC*

## Abstract

Long-term genetic studies utilizing backcross and congenic strain analyses coupled with positional cloning strategies and functional studies identified *Cdkn2a*, *Mtor*, and *Mndal* as mouse plasmacytoma susceptibility/resistance genes. Tumor incidence data in congenic strains carrying the resistance alleles of *Cdkn2a* and *Mtor* led us to hypothesize that drug combinations affecting these pathways are likely to have an additive, if not synergistic effect in inhibiting tumor cell growth. Traditional and novel systems-level genomic approaches were used to assess combination activity, disease specificity, and clinical potential of a drug combination involving rapamycin/everolimus, an *Mtor* inhibitor, with entinostat, an histone deacetylase inhibitor. The combination synergistically repressed oncogenic *MYC* and activated the *Cdkn2a* tumor suppressor. The identification of *MYC* as a primary upstream regulator led to the identification of small molecule binders of the G-quadruplex structure that forms in the NHEIII region of the *MYC* promoter. These studies highlight the importance of identifying drug combinations which simultaneously upregulate tumor suppressors and downregulate oncogenes.

## INTRODUCTION

The majority of human cancers arise in response to exposure to environmental factors and carcinogenic agents that may lead to somatic mutations. Signatures of these mutational processes are often evident in the sequences of cancer genomes^[1]^. Genetic and epigenetic factors also play an important role in determining which exposed individuals will develop tumors. Most tumor susceptibility models in humans and experimental animals have focused on the inherited abnormality of a single gene such as germline mutations of *Rb* or *p53*. These particular single locus lesions are predisposed to tumor formation because they harbor strong “altered function” alleles. However, it is estimated that such strong germline alleles may only account for approximately 2%−14% of human cancers which implies that another paradigm is required to explain the other 86%−98%^[2]^. The individuals in whom these latter cancers arise must either lack a germline genetic component, or tumor development in these individuals represents an inherited trait that may depend on several genes or epigenetic modifiers, in concert with environmental stressors, thus presenting cancer as a complex genetic trait.

Genome wide association studies of cancer development provide a systematic approach to identifying genes that may influence cancer risk^[3]^. Genome-wide linkage studies in genetically uniform strains of mice can provide a window into the more complex genetics associated with human cancers and may be used to model certain patient subpopulations. Thus in 1993, we chose to look at the inheritance of mouse plasmacytoma (PCT) susceptibility alleles associated with genetic variants segregating in immunocompetent backcross mice between *BALB/c* and *DBA/2* strains of mice^[4]^.

## EXPERIMENTAL SYSTEM: IDENTIFICATION OF MOUSE TUMOR SUSCEPTIBILITY PATHWAYS TO TARGET

**Figure 1.**
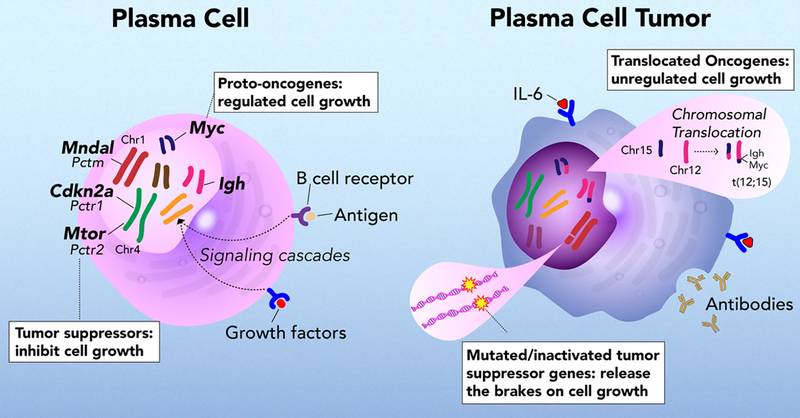
*MYC* regulates cell growth in plasma cells and are dysregulated by translocation in plasma cell tumors^[13,14]^. During plasma cell tumor development, *Cdkn2a* (*p16*) and *Mndal* (interferon inducible gene) expression is low and *Mtor* expression is increased^[6,11,12,15]^

**Figure 2.**
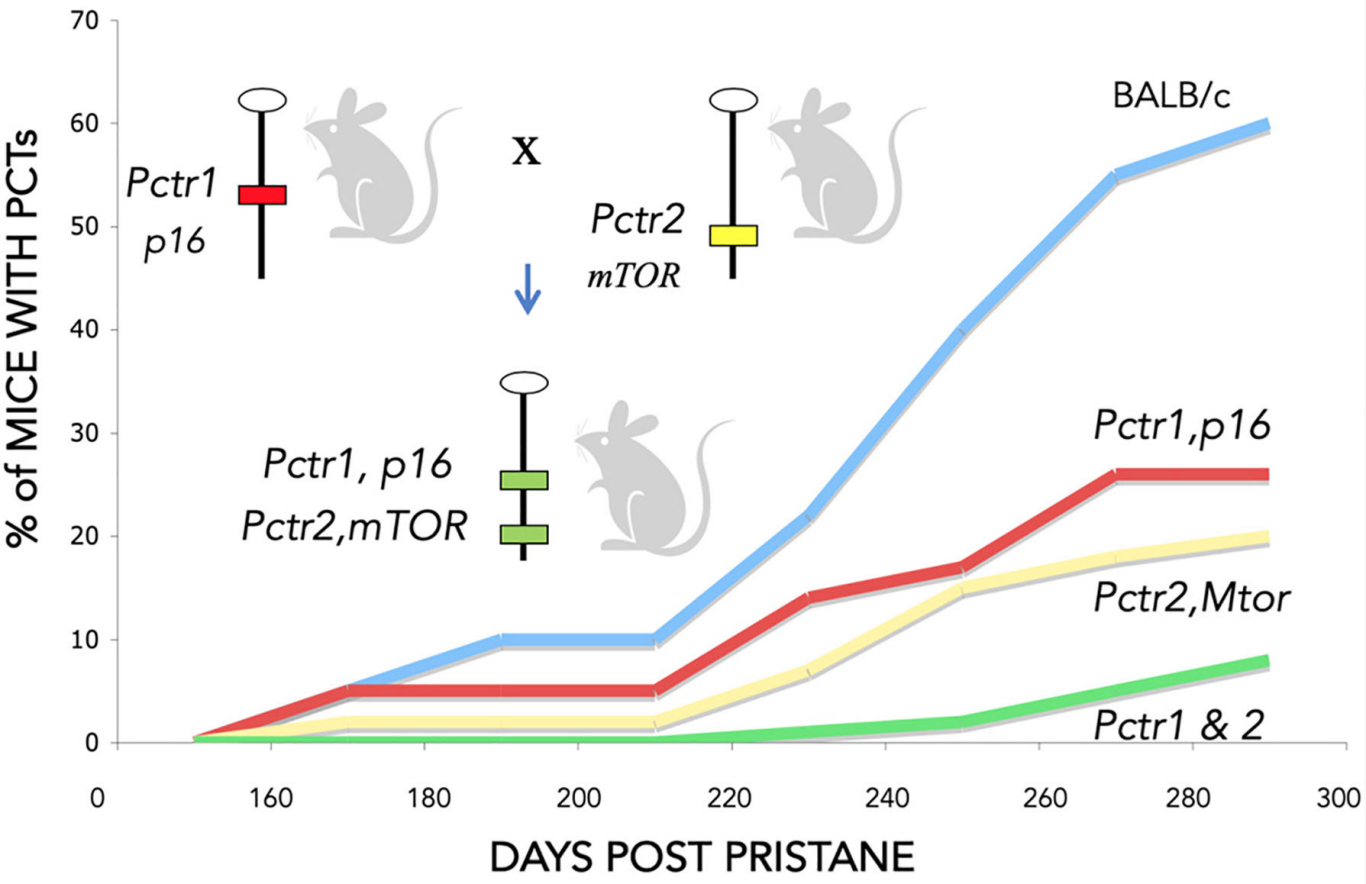
*BALB/c* congenic strains of mice carrying two (*p16* and *Mtor*) *DBA*/*2* plasmacytoma resistance alleles are more resistant to tumor formation than congenics carrying only one of the *Pctr* alleles

Human multiple myeloma (MM) is a clonal proliferative of neoplastic plasma cells in the bone marrow. Mouse plasma cell tumors model certain aspects of these antibody producing neoplasms. Plasmacytomagenesis in *BALB/cAn* mice is a complex genetic trait with 40%−60% penetrance in non-specific pathogen free mice^[5]^. Through our genome-wide mapping studies utilizing genetic crosses with *DBA/2* mice (0% tumor incidence), together with the development and use of a series of *C.D2* congenic strains, coupled with representational difference analysis and positional cloning, we determined that *Cdkn2a* (*p16*), *Mtor*, and *Mndal* contribute to PCT susceptibility and resistance [Figures 1 and 2]^[4–11]^. *Pctr1–2* are localized in non-contiguous, non-overlapping segments of mouse Chr 4, and *Pctm*, a modifier of PCT, on Chr 1. The two *Pctr* loci on Chr 4 are susceptibility loci in *BALB* mice while in *DBA* mice, they are resistance loci as evidenced by backcross and congenic strain analyses. The genes identified for *Pctr1* and *Pctr2* are, *Cdkn2a* (*p16*) and *Mtor*, respectively. The *BALB* alleles of both *p16* and *Mtor* encode efficiency and hypomorphic alleles whose functional activities are much less active than the respective *DBA* alleles. In contrast to *Pctr1* and *Pctr2*, the *Pctm* locus on Chr 1 encodes a resistance allele in *BALB* and a susceptibility allele in *DBA*. In fact, the candidate, *Mndal*, for the *Pctm* locus is deleted in *DBA* mice, but is present and functionally active in *BALB* mice^[12]^.

Compound allelic variation in both coding and promoter sequences, found in *Cdkn2a* [*p16* exon 2: G232A in ANK repeat domain, RREB cis regulatory element (CRE)] and *Mtor* (exon 11: R628C in HEAT repeat domain; MZF1 CRE)^[6,11,16,17]^, contribute to the complex genetics associated with PCT susceptibility in *BALB/c* mice^[4,9]^. Hypomorphic activity of the promoter and coding regions of the *BALB* alleles of both *p16* and *Mtor* is associated with tumorigenesis after exposure to pristane, suggesting that both *Cdkn2a* (*p16*) and *Mtor* can act as tumor suppressors in PCT development in response to stress and in an allele-dependent manner^[11,15–17]^.

*BALB/c* mice are susceptible because they harbor several tumor susceptibility loci that act in concert to produce the susceptible phenotype [Figure 2]. We hypothesize the combination of these relatively subtle allelic defects tip the balance toward both uncontrolled cell growth and a lack of appropriately timed cell death and removal from the cell cycle.

*BALB/c* congenic strains of mice carrying two tumor resistance alleles (*Pctr1* and *Pctr2*) are more resistant than mice carrying only one of the resistance alleles [Figure 2]. This led us to hypothesize that drug combinations targeting these pathways are likely to have a cooperative effect in inhibiting tumor cell growth. The *p16*/*Rb* and *Mtor*/*PI3K* pathways are frequently dysregulated in both mouse plasma cell tumors and in human multiple myeloma^[18–20]^.

## SYNERGISTIC DRUG COMBINATION PHENOCOPIES RESISTANCE ALLELES

The activity of combining *Mtor* and histone deacetylase (HDAC) inhibitors, rapamycin and entinostat respectively, chosen to target the mouse tumor susceptibility pathways (*p16*/*Rb* and *Mtor/PI3K*) was found to be synergistic in limiting the growth of a number of B lineage tumor cell lines, including mouse plasma cell tumors, and the human B cell neoplasms, mantle cell lymphoma, and multiple myeloma^[20]^. We found that combining rapamycin and entinostat elicited responses distinct from a simple combination or the additive effects of the two drugs^[19]^. As such, we developed a rational, unbiased approach to uncover mechanisms of drug synergy for this combination.

### Systems approach

**Figure 3.**
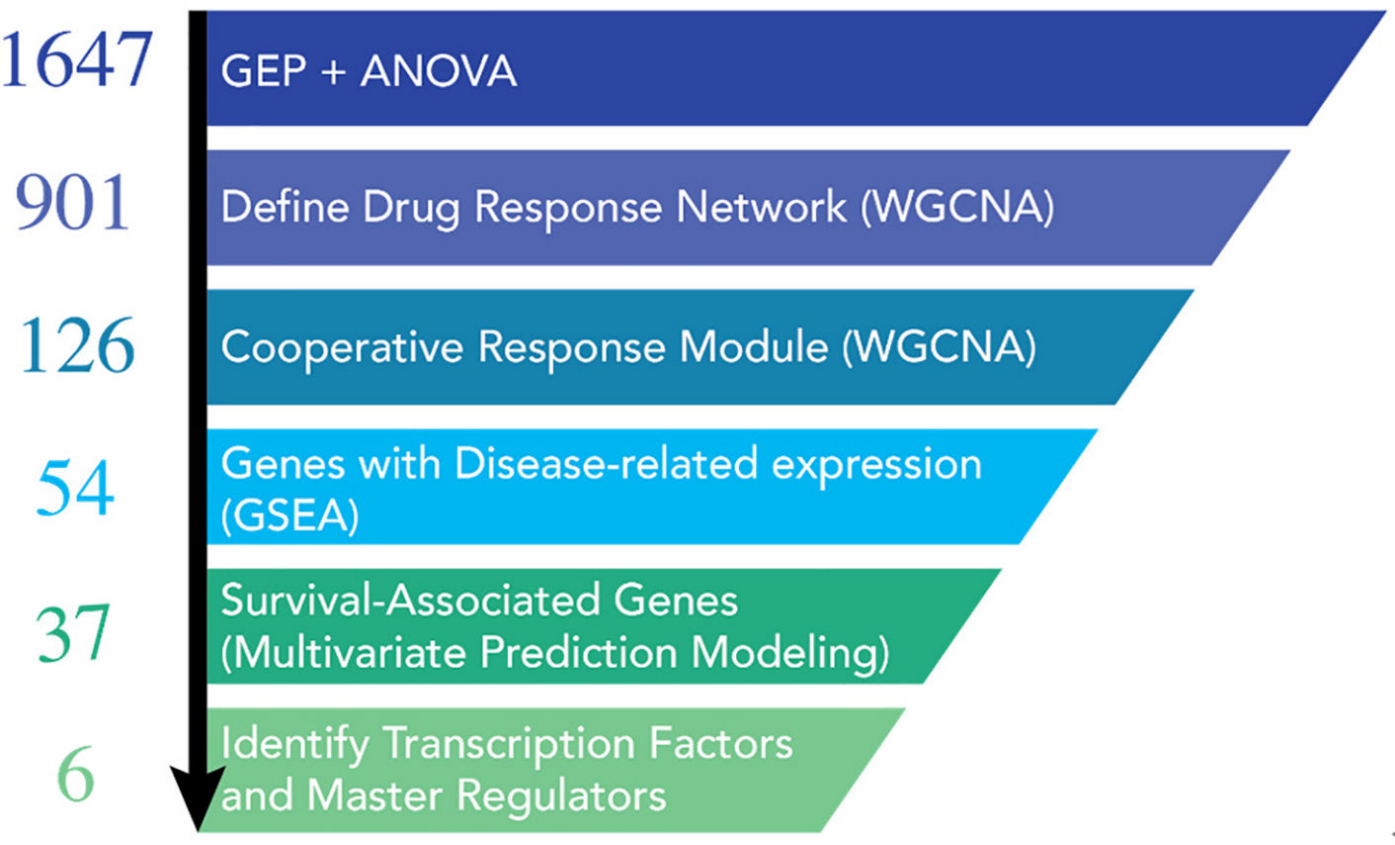
Graphical summary of the systems workflow used to dissect the mechanism of action for the mTOR inhibitor (mTORi) and HDAC inhibitor (HDACi) drug combination^[19]^. Initial ANOVA analysis from our gene expression profiling data started with 1647 differentially expressed genes. Weighted gene co-expression network analyses determined that there were 901 genes in the entire drug response network. Of these 901 genes, 126 genes could be assigned to the drug combination network. These genes were then evaluated for enrichment in myeloma *vs*. normal samples from the same patient (GEO databases) and by multivariate prediction modeling to assess their association with patient survival. 37 disease-specific genes were chosen for further analyses. When the data for the 37 genes (PatentUS2014357660-A1) was evaluated by IPA, 6 master regulators, including *MYC*, *Rb*, and *Cdkn2a* were identified. ANOVA: analysis of variance; GEO: gene expression omnibus; IPA: ingenuity pathway analysis

We evaluated the synergistic activity of combining *Mtor* and HDAC inhibitors at the organismal, cellular, and molecular levels with a cross-disciplinary “systems pharmacology” approach [Figure 3]^[19]^. While the future impact of these specific *Mtor*/HDAC findings is intrinsically linked to the outcome of clinical investigations, there is broader potential for further application and development of our approach. The integration of patient datasets in the identification of a core synergistic response signature offers particular opportunities for the development of companion diagnostics to aid in the clinical development of these combinations. Gene expression-based signatures of cooperative drug responses may prove beneficial for pre-treatment stratification of patients most likely to benefit from a particular drug combination, or as an early response biomarker specific for the combination response and intrinsically linked to expression patterns correlated with improved prognosis. Our approach in this study was enabled by the availability of high-quality, publicly available tumor gene expression datasets from large cohorts of myeloma patients that included either extensive survival annotation or comparisons of healthy *vs*. tumor tissues (GSE4581 and GSE6477)^[21–25]^. The schema depicted below illustrates the approach that we employed to understand the mechanism of drug synergy between the HDAC inhibitor, entinostat and an *Mtor* inhibitor, everolimus^[19]^.

The upstream predictors identified as “activated” from the drug combination by ingenuity pathway analysis (IPA) from the 37 patient-survival associated genes included *Cdkn2a* (*p16*/*p19*), *p53*, and *Rb*. *MYC*, *E2F*, and *TBX2* were predicted as “inhibited” by the combination. The combination worked cooperatively to lower *MYC* protein stability, partially through FBXW7-mediated degradation^[19]^. The combination also worked to increase the activity of the *Rb1*/*Cdkn2a* tumor suppressor pathways^[20]^. The drug combination enhanced the overall survival rate of tumor-bearing *BALB-bclxl* transgenic mice and lowered *MYC* protein levels in tumors of these immunocompetent mice. Our studies in the NCI-60 cell line panel found that most tumors, regardless of their tissue of origin, responded synergistically to the mTORi/HDACi combination. In early molecular classification schemes of multiple myeloma patients based on heirachical clustering of gene expression in myeloma samples, seven clusters were identifed as proliferation (PR), low bone, multiple myeloma SET domain (MMSET), hyperdiploid, cyclin D1 (CD-1), cyclin D2 (CD-2) and avian musculoaponeurotic fibrosarcoma (MAF)^[21]^. Using gene expression data from samples within these same subgroups, we determined a gene score for our 37 drug-responsive genes to predict how many patients would be expected to benefit from combination treatment. Roughly 50% in most subgroups were predicted to benefit; there were two exceptions: all patients in the PR group and only 17% in the CD-2 group were predicted to benefit from the drug combination based on their expression scores for the 37 gene signature^[19]^. This is of course, hypothetical and would need to be tested in a clinical trial. In addition, the drug combination did not have a direct effect on gene expression of genes involved in determining Zhan *et al*.^[21]^’s proliferation index. Cells with mutations in *MYC* residues required for its degradation did not respond to the drug combination^[19]^.

## TARGETING MYC TRANSCRIPTION AND DEGRADATION

Our systems analyses led us to explore a more direct approach to targeting *MYC*. We screened a small molecule microarray library for binders of the G-quadruplex located in the NHEIII region of the *MYC* promoter, and identified a benzofuran-containing molecule, D089, that could stabilize the G4 structure and inhibit *MYC* transcription^[26]^. We demonstrated that D089 inhibited *MYC* with greater affinity than other G4-containing genes (e.g., *RAS*, *VEGF*, *BCL2*, and *Rb1*). The small molecule was relatively potent in inhibiting multiple myeloma cell proliferation but was ineffective in tumor cell lines that had deleted the portion of the *MYC* promoter containing the G4 sequence^[26]^. In subsequent studies, we analyzed a series of analogs to find one, DC-34, that was more potent in its activity against myeloma cells. In these studies, we were able to define an nuclear magnetic resonance structure of the small molecule bound to the G4 structure which should allow structure-guided design of even more potent compounds^[27]^. The discovery of a drug that directly targets *MYC* has been elusive, and thus far there are no approved drugs for this indication. The development of a more direct approach for inhibiting *MYC* activity seems warranted given the overall importance of *MYC* to a wide range of tumor types^[28]^.

**Figure 4.**
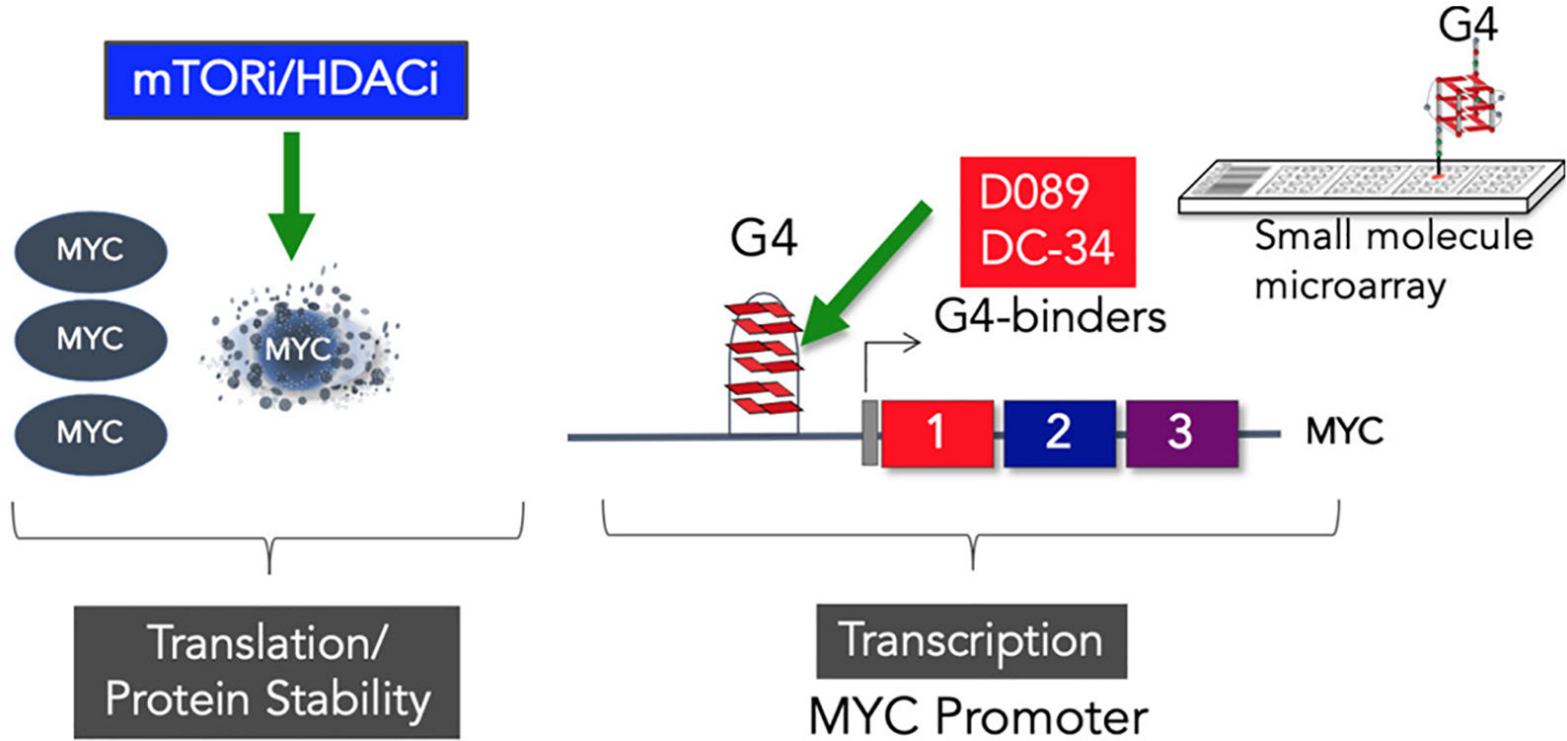
Drug combinations involving mTOR and HDAC inhibitors have a cooperative effect leading to *MYC* protein degradation. Small molecules targeting the G-quadruplex structure in the *MYC* promoter inhibit *MYC* transcription. HDAC: histone deacetylase; mTOR: mechanistic target of rapamycin

Our early work involving retroviral induction studies of mouse PCTs suggest that inhibition of *MYC* alone may not be curative^[29]^. Early induction studies with retroviral vectors clearly showed that overexpression of *MYC* alone could not induce PCTs; but when *MYC* was paired with *RAS* or *RAF*, high incidences of plasma cell tumors could be induced even in PCT-resistant strains of mice^[29]^. These data are in agreement with recent studies indicating that *MYC* mutations are acquired secondary genetic events in myeloma progression^[30]^. A key aspect of the mTORi/HDACi combination is its ability to decrease MYC protein stability; however, in some myeloma cell lines, we have observed a “compensatory” *MYC mRNA* increase with combination treatment, although the steady-state protein level is decreased^[19,20]^. Thus, developing a combinational approach to *MYC* inhibition by inhibiting both transcription and post-translational activity [Figure 4] might be more effective in providing a longer treatment window. Our drug combination studies also highlighted the importance of not only inhibiting *MYC*, but also up-regulating *Rb1*/*Cdkn2a* pathways, again suggesting that a *MYC* G4 stabilizer may not be effective as a single agent^[19,20]^. Combining a *MYC* inhibitor with agents that can upregulate the *RB1*/*CDKN2A* pathways, such as CDK or HDAC inhibitors or other chromatin modifiers, may ultimately be more effective^[31,32]^.

## CONCLUSION

Since the initial sequencing of the human genome in 2001 and the myeloma genome in 2011, there has been a tremendous growth in the generation and availability of high-throughout MM omics datasets^[33–35]^. As a result of this, our knowledge and understanding of genetic underpinnings of MM tumor evolution has seen a similar expansion^[36–38]^. Tumor heterogeneity, both across different patients and between individual subclones within the same patient, has been shown to play an important role in MM disease progression, prognosis, and response to therapeutic treatments^[36,39–41]^.

In a recent study by Maura *et al*.^[42]^ serial whole genome sequencing (WGS) of 30 MM patients was collected and used to determine the chronological order of key driver events that occur during myeloma tumor evolution. In most patients, early driver events such as hyperdiploidy (including the characteristic trisomies of odd chromosomes), immunoglobulin translocation, and chromothripsis tended to precede whole genome duplication, chromoplexy, and point mutation events. In addition to these general patterns of driver event timings, Maura *et al.*^[42]^ also found several examples of co-occurring or mutually exclusive events such as a co-occurrence between t(11;14) and t(14;16) chromosomal translocations and a mutually exclusive pattern of TRAF3 deletions with these same translocations. By combining the data from the 30 patients with an additional 804 patients from the MMRF CoMMpass trial^[43]^, Maura *et al.*^[42]^ were similarly able to detect important driver somatic mutations in MM, including well-known driver genes such as *KRAS*, *NRAS*, and *DIS3*, as well as novel putative driver mutations in genes encoding histone linkers (HIST1H1B, HIST1H1D, HIST1H1E, and HIST1H2BK), and mutations in or near genes involved in nucleosome binding.

Approximately 35%−40% of MM patients have IgH translocations (Chr 14), juxtaposed to an assortment of partners [MMSET (NSD2), FGFR2, MAF, CD-1 and D3 on other chromosomes (4, 6, 8, 11, 16 and 20)]^[44,45]^. In contrast, about 80% of mouse PCTs carry translocations of the IgH locus on mouse Chr 12 juxtaposed to the *MYC* locus on Chr 15^[13]^. Many myeloma mouse genetically engineered models have focused on the dysregulation (overexpression or knock-out) of a particular gene or pathway, most notably, the dysregulation of *MYC* or *BCL2*^[46,47]^, as well as the earlier spontaneous 5T models that have M spikes and develop bone lesions^[48]^. Adoptive B cell transfer mouse models also provide a novel approach to study MM pathogenesis^[49]^. Vlummens *et al.*^[50]^ comprehensively reviewed numerous murine models, ranging from xenografts to immunocompetent spontaneous and transgenic models, for studying both the etiology and pathogenesis of MM. More recently, Rajagopalan *et al*.^[51]^ generated a Nras^LSL Q61R/+^ mouse which takes advantage of crossing Vk*MYC mice to mice harboring a Q61R *NRAS* mutation (as found in WGS studies of myeloma)^[35,42,46]^. This rapid model also develops both bone lesions and M spikes.

In contrast, the focus of our studies has been on genetically inherited alleles of genes in immunocompetent strains of mice that predispose the mice to peritoneal plasmacytoma development. In the past several years, more than 17 risk loci for multiple myeloma susceptibility have now been mapped to unique regions of the human genome^[52–57]^. The one gene in common with our studies is the *Cdkn2a* locus; it is a tumor susceptibility gene in both mouse plasma cell tumors by genetic linkage studies^[4,6,15]^ and in genome-wide association studies (GWAS) in human multiple myeloma^[52]^. Much progress has been made in understanding the omics of myeloma through GWAS^[52–57]^, eQTL^[58]^, and WGS^[35,42]^ studies of myeloma patient samples. These studies have helped to identify new targets for intervention of myeloma disease progression and form the basis for developing companion diagnostics for drug treatments.

In our studies, we have viewed cancer treatment through the lens of cancer as a complex genetic trait by using pristane-induced mouse PCT as the model^[4]^. A goal in the molecular identification of these susceptibility/resistance genes has been to uncover the signaling pathways that are involved in promoting or controlling B cell neoplasia and to understand how these pathways may act in concert to contribute to or limit tumor progression. Tumor incidence data in congenic strains of mice, constructed to harbor different combinations of resistance alleles^[5,9]^, led to the hypothesis that drug combinations affecting these pathways are likely to have at least an additive, if not synergistic effect in inhibiting tumor cell growth. We investigated experimental therapeutic approaches to target myeloma; that led to the twin goals of upregulating tumor suppressor activities and downregulating oncogenic processes simultaneously.

Our initial preclinical studies focused on *Mtor* inhibition, through targeting *Mtor* kinase activity, coupled with HDAC inhibition, which inhibits histone deacetylation. HDAC inhibitors can target a broad spectrum of genes involved in chromatin modification, including those that regulate the *Rb1* and *p16* pathways. Our mechanistic analysis of the successful targeting of these two pathways, which induced synergistic anti-tumor activity in susceptible tumors, identified *MYC* as an important upstream driver regulated by the combined pathways through their cooperative effects on *MYC* protein degradation. Drug combinations targeting the two signaling pathways (*Cyclin D*/*CDK*/*Cdkn2a*/*Rb* and *PI3K*/*AKT*/*Mtor*) identified by our genetic analysis of PCT susceptibility, were indeed synergistic in their activity, not only for myeloma, but also a variety of tumor types as shown in their broad synergistic activity in the panel of NCI-60 cell lines. In fact, the only NCI-60 cell line for which this combination was antagonistic had a mutation in *FBXW7* which is involved in *MYC* protein degradation^[19]^.

*MYC* is often overexpressed and/or dysregulated in cancer, including mouse PCT, as well as human myeloma and Burkitt’s lymphoma^[30,59,60]^. *MYC* has often been considered an undruggable target, yet many researchers are pursuing a number of avenues to downregulate *MYC*, including drug combinations such as the one described above to target post-translational steps, such as protein stability. In addition, *MYC*’s transcription factor activity requires dimerization with its binding partner MAX (MYC-associated factor x), and many efforts have focused on interrupting this complex to downregulate its transcription factor activity^[61]^. Furthermore, *JQ1*, a BET (bromodomain and extra-terminal domain) inhibitor, can also inhibit *MYC* transcription, as well as other pathways^[62]^. We and others have focused on interfering with *MYC* gene transcription by an alternative inhibitory mechanism involving small molecules that stabilize complex *DNA* structures (G-quadruplexes) which form transiently in the *MYC* promoter^[26,27,63,64]^.

Our studies to identify cooperative targets of mTORi/HDACi inhibition have: (1) provided a basic approach for broader application to identify potential biomarkers of drug combinations utilizing weighted gene coexpression network analyses combined with gene set enrichment analyses of survival annotated patient datasets; (2) identified upstream regulators/drivers of drug responses leading to a mechanistic understanding of how the combination is acting (upregulation of tumor suppressive pathways (*Rb1* and *p16*) and downregulation of oncogenic pathways (*MYC* and *E2F1*), leading to *MYC* degradation; and (3) demonstrated that cell lines carrying mutations in *FBXW7* or surrounding *MYC* residues Thr58,Ser62, involved in *MYC* degradation are not likely to respond to the combination of rapamycin/everolimus and entinostat. While *MYC* is known to be deregulated in a majority of cancers, its direct drug targeting has been elusive. We hope that our work to identify and develop a new class of compounds targeting *MYC* transcription will lead to new pharmacological agents for *MYC* inhibition. Our studies suggest that it would be clinically useful if these inhibitors were coupled with drugs that simultaneously upregulate tumor suppressors. Our studies are designed to provide the pre-clinical rationale and evidence of synergistic mechanisms required to advance candidate combinations for preclinical assessment in patient-derived cells and eventually in clinical study.
